# Acute respiratory infection emergency access in a tertiary care children hospital in Italy, prior and after the SARS‐CoV‐2 emergence

**DOI:** 10.1111/irv.13102

**Published:** 2023-03-20

**Authors:** Marta Ciofi degli Atti, Caterina Rizzo, Carmen D'Amore, Lucilla Ravà, Antonino Reale, Maria Antonietta Barbieri, Paola Bernaschi, Cristina Russo, Alberto Villani, Carlo Federico Perno, Massimiliano Raponi

**Affiliations:** ^1^ Clinical Pathways and Epidemiology Unit, Medical Direction Bambino Gesù Children's Hospital, IRCCS Rome Italy; ^2^ Department of Translational Research on New Technologies in Medicine and Surgery University of Pisa Pisa Italy; ^3^ Pediatric Emergency Department Bambino Gesù Children's Hospital, IRCCS Rome Italy; ^4^ Microbiology and Diagnostic Immunology Unit Bambino Gesù Children's Hospital, IRCCS Rome Italy; ^5^ Medical Direction Bambino Gesù Children's Hospital, IRCCS Rome Italy

**Keywords:** acute respiratory infections, children, COVID‐19 pandemic, emergency visit, viral infections

## Abstract

**Background:**

The COVID‐19 pandemic has changed the epidemiology of acute respiratory infections (ARIs) in children. The aims of the present study were to describe the epidemiological trend of ARI emergency visits and virology results prior and after the SARS‐CoV‐2 emergence and to estimate the association of ARI emergency department (ED) visits with respiratory viruses.

**Methods:**

This study was conducted at the Bambino Gesù Children's Hospital, a tertiary care children's hospital in the Lazio Region, Italy. The demographic and clinical information of children who accessed the ED and were diagnosed with ARI from January 1, 2018 to June 30, 2022 was retrospectively extracted from the electronic health records. The observed temporal trends in viruses diagnosed from respiratory samples were compared with the number of ARI ED visits over the same period through a multivariable linear regression model.

**Results:**

During the study period, there were 72,959 ED admissions for ARIs and 33,355 respiratory samples resulted positive for viruses. Prior to the pandemic, respiratory syncytial virus (RSV) and influenza had a clear seasonal pattern, which was interrupted in 2020. In 2021–2022, RSV reached the highest peak observed during the study period, whereas influenza activity was minimal. The peaks of ARI ED visits corresponded to peaks of influenza, RSV, and rhinovirus in the 2018–2019 and 2019–2020 seasons, to SARS‐CoV‐2 and rhinovirus in 2020, and to RSV and parainfluenza in 2021–2022.

**Conclusions:**

ARI resulting in ED visits should be included in the ARI disease burden measurement for a more accurate measure of the impact of preventive measures.

## 1 INTRODUCTION

Acute respiratory infections (ARIs) are the most common cause of pediatric emergency visits^1^ and are most frequently caused by viruses, such as rhinovirus, enterovirus, respiratory syncytial virus (RSV), influenza virus, parainfluenza virus, bocaviruses, adenoviruses, and metapneumovirus.^2^, ^3^, ^4^, ^5^

Italy was the first country to be affected by COVID‐19 in Europe, with the first native patient diagnosed on February 20, 2020. National response actions to contain the pandemic upgraded from strict social distancing measures in 11 municipalities in Northern Italy, on February 23, 2020, to national social distancing and school closures on March 4, 2020 and culminated with the national lockdown from March 11 to May 4, 2020.^6^ From October 2020 to June 2021, social distancing measures were progressively reduced and adapted according to the evolution of the COVID‐19 pandemic.

The COVID‐19 pandemic has deeply changed the epidemiology of ARIs in children; public health measures introduced to prevent the pandemic affected the transmission of respiratory viruses,^7^, ^8^ interfered with the seasonality of childhood respiratory diseases and reduced ED visits and hospitalizations due to ARIs.^7^, ^9^, ^10^ With the reduction of social isolation measures, an unexpected RSV seasonality was observed in 2021–2022, with an earlier peak of shorter duration in comparison with pre‐pandemic seasons.^11^, ^12^ Regression models are frequently used to determine what proportion of the outcome of interest (such as outpatient visits, ED visits or hospitalizations) might be attributable to specific infections, such as influenza.^13^, ^14^, ^15^ The aims of the present study were to describe the epidemiological trend of ARI emergency visits and virology results in a tertiary care children hospital and to estimate the association of ARI ED visits with respiratory viruses, from January 2018 to June 2022.

## 2 METHODS

### 2.1 Setting

This study was conducted at the Bambino Gesù Children's Hospital (IRCCS Ospedale Pediatrico Bambino Gesù, hereafter OPBG), a 607‐bed tertiary care academic hospital located in the Lazio Region, Italy. The emergency department (ED) provides free urgent medical care to the pediatric population on a 24/7 basis; ED admissions were 85,012 in 2018, 89,558 in 2019, 62,010 in 2020, 79,624 in 2021, and 38,191 in the first semester 2022.

### 2.2 Data collection

The demographic and clinical information of children who accessed the ED and were diagnosed with ARI from January 1, 2018 to June 30, 2022 was retrospectively extracted from the electronic health records of OPBG.

In detail, information on ED visits was collected from the OPBG Healthcare Emergency Information System (HEIS), which includes patient demographics, ICD‐9‐CM diagnosis, and status at discharge (i.e., hospitalized or discharged at home). ARI was defined according to the ICD‐9 CM diagnosis at discharge (see supporting information S1).

Information on virology results of respiratory samples (i.e., naso‐pharyngeal swabs, tracheal swabs, and/or broncho‐alveolar lavages) obtained in the same time period from OPBG patients was derived from the Hospital Electronic Laboratory Information System. Respiratory samples testing positive for the same virus within 3 months were excluded from the analysis.

### 2.3 Laboratory testing

Respiratory samples were tested with a multiple target RT‐PCR assays able to simultaneous detection of 18 respiratory viruses (including RSV A and B, influenza virus A and B, human coronavirus OC43, 229E, NL‐63 and HUK1, adenovirus, human rhinovirus [hRV], parainfluenza virus 1‐2‐3‐4, human metapneumovirus‐hMPV, human bocavirus‐hBoV, and enterovirus) through commercial multiplex RT‐PCR kit (Allplex™ Respiratory Panel Assay Seegene Korea).

Nucleic acids were extracted by using a one Step Combination extraction and PCR set up starting from a 200 μL sample of respiratory samples directly loaded to an automated instrument by using a ready‐to‐use extraction reagent cassettes (StarMag universal cartridges on Seegene Starlet) and amplified on CFX96TM Real‐Time PCR Detection System (BioRad Laboratories).

Starting from 2020, SARS‐CoV‐2 molecular assay or SARS‐CoV‐2 antigen tests were also performed.

### 2.4 Statistical analysis

The number of ED admissions and positive tests by year, week, and age class were summarized as counts and percentages; positive tests were also described by type of virus; significance in trend was tested using Cochrane Armitage test.

The observed temporal trends in viruses diagnosed from respiratory samples were compared with the number of ARI ED visits over the same time period. The latter was used as the dependent variable of a multiple linear regression. The independent variables were the weekly number of viral laboratory results. The expected number of ARI ED visits *Yj* in a week period *j* was the following:

Yj=c+c+∑∝jLiJ

where *Lij* is the number of laboratory reports for virus *i* in week *j*, *αi* is the regression coefficient for virus *i* used to estimate the number of ARI ED admissions associated with each virus, and *c* is a constant representing the background number of ARI ED visits that were not explained by viruses included in the model.

A multivariable linear regression was performed and included all the viruses as explanatory variables. The significance of each virus was assessed, and if the *p*‐value was >0.2 or the variable was significant but the resulting coefficient was negative (which is biologically implausible), the variable was discarded from the model. The validity of the final model was assessed in terms of the proportion of the variation it explained (R2), the significance of the joint relationship between the observed and independent variables in each model (i.e., the probability that R differed significantly from zero), and the impact of changes in the model specification. We also computed specific multivariable linear regression models for age classes (≤1 years, 1–4 years, 5–9 years, > = 10 years). The attribution of the ARI rate to each virus was generated by multiplying each regression coefficient by its corresponding virus count.

All statistical analyses were performed using STATA, Statistical Software: Release 17 (StataCorp LP, College Station, TX).

## 3 RESULTS

From January 2018 to June 2022, there were 72,959 ED visits for ARIs (Table 1). In 2020, the number of ARI ED visits decreased by 30.9% compared with 2019 (12,923 vs. 18,717), while in 2021, ARI ED visits increased by 24.6% compared with 2020 (16,099 vs. 12,923) and approached the number observed in 2018.

**TABLE 1:** ED visits and hospitalizations due to ARI by time period and age group; OPBG, January 2018 to June 2022.

	2018		2019		2020		2021		2022^a^		Total		*P‐value for trend ED visits*	*P‐value* *for trend hospitalizations*
Age	N. visits (%)	*N. hospitalizations (% of visits)*	N. visits (%)	*N. hospitalizations (% of visits)*	N. visits (%)	*N. hospitalizations (% of visits)*	N. visits (%)	*N. hospitalizations (% of visits)*	N. visits (%)	*N. hospitalizations (% of visits)*	N. visits (%)	*N. hospitalizations (% of visits)*		
**<1 year**	4517 (26.5)	*1201 (26.6)*	4586 (24.5)	*1175 (25.6)*	2666 (20.6)	*660 (24.8)*	3611 (22.4)	*745 (20.6)*	1594 (19.5)	*160 (10.0)*	16 974 (23.3)	*3941 (23.2)*	*<0.001*	*<0.001*
**1–4 years**	8085 (47.5)	*579 (7.2)*	8743 (46.7)	*589 (6.7)*	5476 (42.4)	*435 (7.9)*	8923 (55.4)	*497 (5.6)*	4215 (51.5)	*192 (4.6)*	35 442 (48.6)	*2292 (6.5)*	*<0.001*	*<0.001*
**5–9 years**	2748 (16.1)	*258 (9.4)*	3324 (17.8)	*266 (8.0)*	2662 (20.6)	*213 (8.0)*	1873 (11.6)	*151 (8.1)*	1322 (16.1)	*86 (6.5)*	11 929 (16.4)	*974 (8.2)*	*<0.001*	*0.005*
**> = 10 years**	1679 (9.9)	*270 (16.1)*	2064 (11.0)	*302 (14.6)*	2119 (16.4)	*319 (15.1)*	1692 (10.5)	*321 (19.0)*	1060 (12.9)	*128 (12.1)*	8614 (11.8)	*1340 (15.6)*	*<0.001*	0.7
**Total**	**17 029 (100.0)**	***2308 (13.6)***	**18 717 (100.0)**	***2332 (12.5)***	**12 923 (100.0)**	***1627 (12.6)***	**16 099 (100.0)**	***1714 (10.6)***	**8191 (100.0)**	***566 (6.9)***	**72 959 (100.0)**	***8547 (11.7)***	*<0.001*	*<0.001*

*Note*: Bold emphasis is used to evidentiate the titles of the rows and columns and the total numbers and proportions. Italics emphasis is used to evidentiate that numbers and proportions of hospitalizations are a subgroup of ED visits. *P*‐values are also reported in italics.

Abbreviations: ARI, acute respiratory infection; ED, emergency department; OPBG, Ospedale Pediatrico Bambino Gesù.

^a^ January to June.

Children aged 1–4 years accounted for 48.6% (*n* = 35 442) of the ED visits, followed by those aged <1 year (*n* = 16 974, 23.3%), 5–9 years (*n* = 11.929, 16.4%), and > = 10 years (*n* = 8614, 11.8%); the proportion of ED visits significantly decreased in patients aged <1 year old, whereas it increased in children aged 1–4 and > = 10 years old.

Of the total children visited for ARI, 11.7% (*n* = 8547) were hospitalized; the highest proportion of hospitalizations was seen in infants <1 year of age (23.2%), followed by children ≥ 10 years (15.6%) (Table 1). The proportion of children hospitalized for ARI significantly decreased from 13.6% (*n* = 2308) of ARI ED visits in 2018 to 6.7% in 2022 (566; *p* < 0.001); reduction in hospitalizations for ARI was statistically significant in all age classes up to 10 years of age.

Out of a total of 30,355 respiratory samples that resulted positive for viruses, the most frequently confirmed virus was SARS‐CoV‐2 (38% of tests; *n* = 11 496), which emerged in 2020 and increased by year, followed by rhinovirus (20%; *n* = 6092), RSV (11%; *n* = 3396), influenza (6%; *n* = 1674), parainfluenza (5%; *n* = 1636), adenovirus (5%; *n* = 1390), and bocavirus (4%; *n* = 1178). The distribution of respiratory viruses diagnosed from respiratory samples significantly varied by year (Table 2).

**TABLE 2:** Distribution of viral respiratory infections by time period; OPBG, January 2018 to June 2022.

Year	2018		2019		2020		2021		2022^a^		Total		*P‐value for trend*
Respiratory virus	*N*	%	*N*	%	*N*	%	*N*	%	*N*	%	*N*	%	
SARS‐CoV‐2	‐	‐	‐	‐	2159	43.8	3737	46.4	5600	78.0	**11 496**	**37.9**	*<0.001*
Rhinovirus	1828	35.9	1809	35.5	998	20.2	956	11.9	501	7.0	**6092**	**20.1**	*<0.001*
VRS	990	19.4	937	18.4	382	7.7	988	12.3	99	1.4	**3396**	**11.2**	*<0.001*
Influenza	401	7.9	588	11.5	588	11.9	9	0.1	88	1.2	**1674**	**5.5**	*<0.001*
Parainfluenza	440	8.6	354	6.9	86	1.7	596	7.4	160	2.2	**1636**	**5.4**	*<0.001*
Adenovirus	345	6.8	399	7.8	196	4.0	279	3.5	171	2.4	**1390**	**4.6**	*<0.001*
Bocavirus	313	6.1	276	5.4	164	3.3	291	3.6	134	1.9	**1178**	**3.9**	*<0.001*
Coronavirus	281	5.5	262	5.1	173	3.5	311	3.9	131	1.8	**1158**	**3.8**	*<0.001*
Human rhinovirus/enterovirus	0	0.0	0	0.0	15	0.3	699	8.7	129	1.8	**843**	**2.8**	*<0.001*
Enterovirus (HEV)	321	6.3	277	5.4	110	2.2	27	0.3	57	0.8	**792**	**2.6**	*<0.001*
Human metapneumovirus	174	3.4	197	3.9	62	1.3	162	2.0	105	1.5	**700**	**2.3**	*<0.001*
Total	**5093**	**100.0**	**5099**	**100.0**	**4933**	**100.0**	**8055**	**100.0**	**7175**	**100.0**	**30355**	**100.0**	*<0.001*

*Note*: Bold emphasis is used to evidentiate the titles of the rows and columns and the total numbers and proportions. Italics is used for *P*‐values.

Abbreviation: OPBG, Ospedale Pediatrico Bambino Gesù.

^a^ January to June.

**FIGURE 1:**
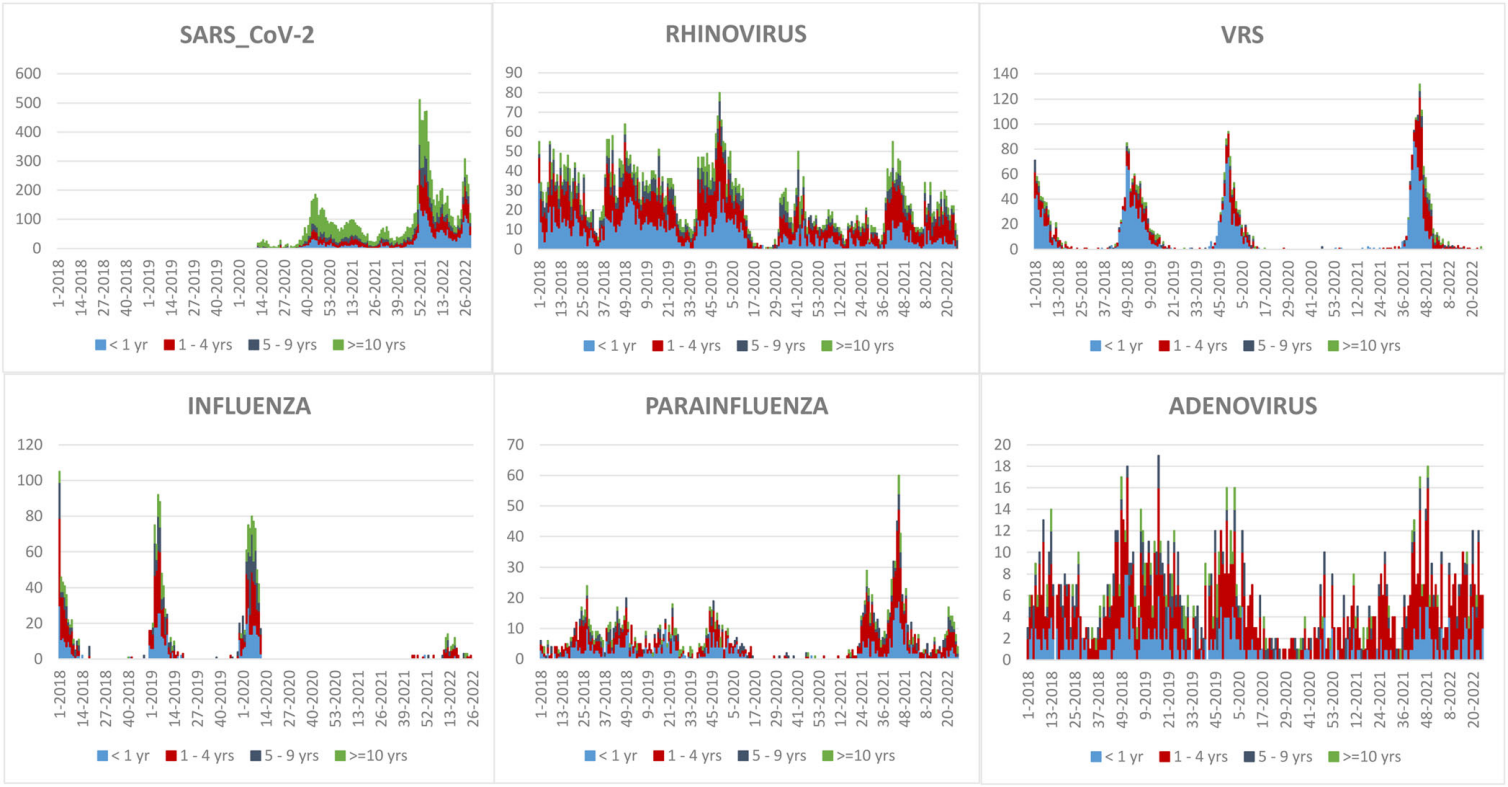
Weekly trend of the six most frequent confirmed viruses; Ospedale Pediatrico Bambino Gesù (OPBG), January 2018 to June 2022.

Figure 1 shows the weekly trend of the top six viruses in terms of frequency diagnosed in the study period (SARS‐CoV‐2, rhinovirus, RSV, influenza, parainfluenza, and adenovirus). All viral respiratory infections sharply decreased during 2020. Prior to the pandemic, RSV and influenza had a clear seasonal pattern, which was interrupted in 2020. In 2021–2022, RSV and Parainfluenza reached the highest peak observed during the study period; in 2020–2021 and 2021–2022, there was a minimal influenza activity. Rhinovirus and adenovirus had less variations by year.

**FIGURE 2:**
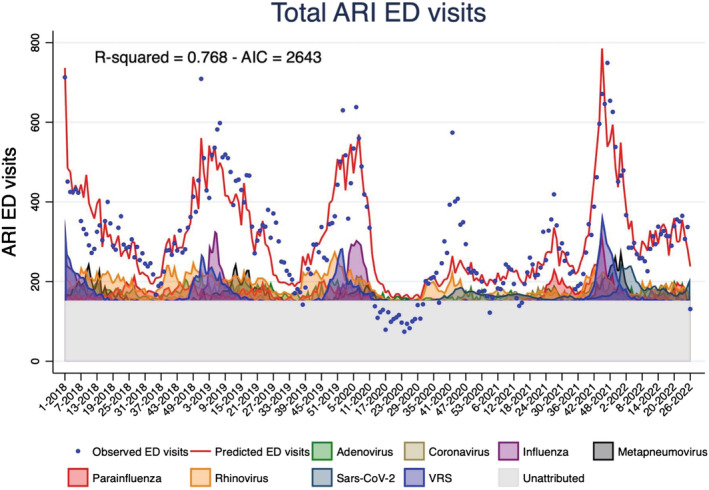
Multivariable linear regression model—weekly emergency department (ED) acute respiratory infection (ARI) visits; Ospedale Pediatrico Bambino Gesù (OPBG), January 2018 to June 2022.

Observed ARI ED visits were in line with those predicted by the model from January 2018 to March 2020 and from May 2021 onwards (Figure 2). From March to July 2020, the model predicted a higher number of ARI ED visits than observed, while from October to December 2021, the observed ARI ED visits were more frequent than expected. The peaks of ARI ED visits corresponded to peaks of influenza, RSV, and rhinovirus in 2018–2019 and 2019–2020 seasons, to SARS‐CoV‐2 and rhinovirus in 2020, and to RSV and parainfluenza in 2021–2022.

**FIGURE 3:**
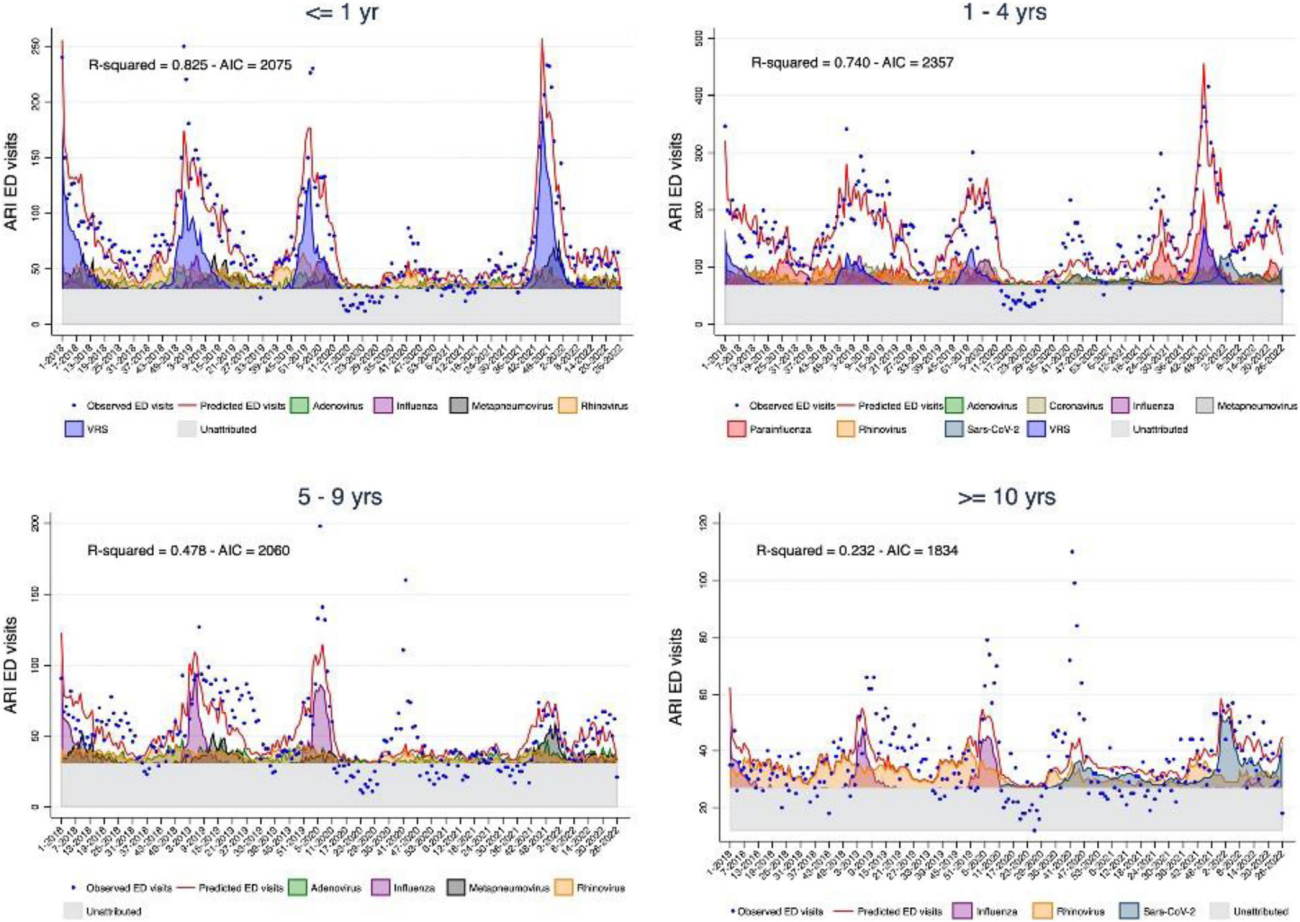
Multivariable linear regression models ‐ weekly emergency department (ED) acute respiratory infection (ARI) visits by age group; Ospedale Pediatrico Bambino Gesù (OPBG), January 2018 to June 2022 by age group.

The best goodness‐of‐fit of the models by age group was in infants ≤1 year of age, where the model peaks of ARI ED visits clearly reflect RSV circulation (Figure 3). When considering all age groups, the model attributed 14% of the ARIs presenting at the ED to rhinovirus, 8% to RSV and adenovirus, 6% to influenza and metapneumovirus, 5% to parainfluenza and coronaviruses other than SARS‐CoV‐2, and 3% to SARS‐CoV‐2 (supporting information S2). The proportion of ARI ED visits attributed to viruses sharply decreased by age; in infants <1 year, 20% of the visits were attributed to RSV and 15% to rhinovirus; in 1‐ to 4‐year‐old children, the most frequent viruses impacting on ARI ED visits were parainfluenza (11%) and rhinovirus (10%); in 5‐ to 9‐year‐old children were influenza (14%), adenovirus (12%), and rhinovirus (11%); and in ≥10‐year‐old children was rhinovirus (16%).

## 4 DISCUSSION

Our study showed a clear pattern of ARI incidence and seasonality that follows the dynamics of the COVID‐19 pandemic. The reduction in ARI cases and circulation of respiratory viruses during the pandemic period can be explained by the impact of public health and social distancing measures on overall respiratory disease transmission, as observed by other surveillance systems internationally.^7^, ^10^, ^16^, ^17^

The viruses associated with ARI in children were consistent with those reported by other studies in several countries worldwide,^4^, ^5^, ^18^, ^19^ but the epidemiology of various viruses was affected by the pandemic in different ways.

In our study, it was possible to identify the abrupt and early end of the 2019/2020 influenza wave, as observed by Influnet surveillance in Italy^20^ and in other European countries.^21^ The data also clearly show that in 2020/2021, there was no normal flu season,^22^ and the low influenza activity continued in 2021/2022 season, despite suspension of strict pandemic control measures, such as school closure. Possible reasons include the effectiveness of mask wearing and social distancing on preventing transmission of influenza and the reduction of national and travels that facilitate its spreading.^23^

We confirmed that RSV is the leading cause of ARI in young children and has a high seasonality,^8^ which was disrupted by the COVID‐19 pandemic. After the reduction of cases in 2020, the peak of RSV incidence in 2021–2022 was striking. As reported by other authors, this may be due to the increasing number of children that were naive for RSV, due to the lack of RSV circulation in the population during the strict social distancing measures for the SARS‐CoV‐2 pandemic.^8^ We documented a similar picture for parainfluenza, which in 2021–2022 reached the highest peak observed since 2018. A resurgence of parainfluenza was observed also in China, in a nationwide study that reported its increase in the <18‐year‐old group, when schools were re‐opened in most provinces in 2020–2021.^24^

In this study, the regression model estimates of ARI visits corresponded closely with the observed weekly numbers in children <5 years of age, where ARIs are a leading cause of morbidity and mortality.^25^ We documented that almost 60% of the ARIs in all the age groups of children can be attributed to a viral infection and that different viruses play a role in different age groups. RSV was common in infants under the age of 1 year, whereas influenza was responsible for most ED admissions in children aged 6–9 years. Our results were also consistent with a recent modeling study reporting that individuals aged 5–14 years were the most affected by influenza virus compared with other age groups.^26^ This finding is of particular interest because from the 2020/2021 season, the Ministry of Health has recommended influenza vaccination in healthy children aged between 6 months and 6 years, in Italy.^27^ The recommendation was based on the high incidence of cases of influenza‐like illness (ILI) (not laboratory confirmed, though) reported to the national influenza surveillance system (InfluNet) in the 0–5 age group.^28^ Our data on ED admissions by age group and respiratory virus could be very useful in supporting national vaccination strategies. During the entire study period, the proportion of ED visits attributed to SARS‐CoV‐2 was 3%, despite that it was the most frequently isolated virus from respiratory samples. This may be due to the hospital policy of testing for SARS‐CoV‐2 all patients at hospital admission and to the possibility that Covid‐19 cases were classified with ICD9 codes other than those included in the ARI definition adopted in this study. The ARI rate that was not attributed to viruses included in the models may be caused by other viruses or to bacterial infections.

Compared with the available literature, our research has several strengths. First, all the analyzed patients had received a diagnosis of ARI by pediatricians of a third level teaching children's hospital. Secondly, we provided an extended analysis of viruses causing ARI in children. On the other hand, the retrospective monocentric design and the lack of correlation between detected pathogens and clinical severity were among the potential drawbacks of our study.

ARI causes a significant health burden in terms of ED visits and hospitalizations among children. In this study, 20% of ED visits were due to ARI, and 11% resulted in hospitalization. Moreover, due to the high number of ED admissions and hospitalizations concentrated in few weeks during the winter season, the burden of ARIs affects the whole hospital pathway. ARIs resulting in ED visits should then be included in the ARI disease burden measurement to more accurately evaluate the impact of vaccines, immunoprophylaxis therapies, and other preventive measures.

## AUTHOR CONTRIBUTIONS

MCDA and CR conceived the study; MCDA, CR, and CD wrote the manuscript; LR performed statistical analysis; AR, MAB, PB, CR, AV, CFP, and MR reviewed the manuscript. All the authors reviewed the final version of the manuscript and agreed to be accountable for the content of the work.

## CONFLICT OF INTEREST

The authors declare that they have no competing interests.

### ETHICS APPROVAL STATEMENT

The study was approved by the Ethics Committee of the Bambino Gesù Children's Hospital.

### PEER REVIEW

The peer review history for this article is available at https://publons.com/publon/10.1111/irv.13102.

## PATIENT CONSENT STATEMENT

Considering the retrospective study design, written informed consent was not deemed necessary.

## PERMISSION TO REPRODUCE MATERIAL FROM OTHER SOURCES

Not applicable.

## Supporting information


**Supplementary Material 1.** ICD 9‐CM diagnosis at ED discharge for ARI

**Supplementary Material 2:** Percentages of ARI ED virus attributed to different respiratory virus counts by age class

## Data Availability

The data that support the findings of this study are available from the corresponding author upon reasonable request.
