# Supplementary material for: Acute respiratory infection emergency access in a tertiary care children hospital in Italy, prior and after the SARS‐CoV‐2 emergence
Source: Influenza Other Respir Viruses. 2023 Mar 20;17(3):e13102. doi: 10.1111/irv.13102 (PMC10026100; doi:10.1111/irv.13102)
Supplement: Supplementary file 1 — Supplementary Material 1. ICD 9‐CM diagnosis at ED discharge for ARI [file IRV-17-e13102-s001.docx]

**Supplementary Material 1**. ICD 9-CM diagnosis at ED discharge for ARI

| **ICD-9 CM code and description** |
| --- |
| 460 ACUTE NASOPHARYNGITIS |
| 461 AC SINUSITIS |
| 4610 AC MAXILLARY SINUSITIS |
| 4611 AC FRONTAL SINUSITIS |
| 4612 AC ETHMOIDAL SINUSITIS |
| 4613 AC SPHENOIDAL SINUSITIS |
| 4618 OTHER ACUTE SINUSITIS |
| 4619 ACUTE SINUSITIS NOS |
| 462 ACUTE PHARYNGITIS |
| 463 ACUTE TONSILLITIS |
| 4640 AC LARYNGITIS |
| 46400 AC LARYNGITIS W/O OBST |
| 46401 AC LARYNGITIS W OBSTRUCT |
| 46410 AC TRACHEITIS NO OBSTRUC |
| 46411 AC TRACHEITIS W OBSTRUCT |
| 4642 AC LARYNGOTRACH |
| 46420 AC LARYNGOTRACH NO OBSTR |
| 46421 AC LARYNGOTRACH W OBSTR |
| 4643 AC EPIGLOTTITIS |
| 46430 AC EPIGLOTTITIS NO OBSTR |
| 46431 AC EPIGLOTTITIS W OBSTR |
| 4644 CROUP |
| 4659 ACUTE URI NOS |
| 4660 ACUTE BRONCHITIS |
| 46611 ACU BRONCHOLITIS D/T RSV |
| 46619 ACU BRNCHLTS D/T OTH ORG |
| 4800 ADENOVIRAL PNEUMONIA |
| 4801 RESP SYNCYT VIRAL PNEUM |
| 4802 PARINFLUENZA VIRAL PNEUM |
| 4808 VIRAL PNEUMONIA NEC |
| 4809 VIRAL PNEUMONIA NOS |
| 481 PNEUMOCOCCAL PNEUMONIA |
| 48283 PNEUMO OTH GRM-NEG BACT |
| 48289 PNEUMONIA OTH SPCF BACT |
| 4829 BACTERIAL PNEUMONIA NOS |
| 4830 PNEU MYCPLSM PNEUMONIAE |
| 4838 PNEUMON OTH SPEC ORGNSM |
| 485 BRONCHOPNEUMONIA ORG NOS |
| 486 PNEUMONIA, ORGANISM NOS |
| 4870 INFLUENZA WITH PNEUMONIA |
| 4871 FLU W RESP MANIFEST NEC |
| 4878 FLU W MANIFESTATION NEC |
| 7806 FEVER |
| 78606 TACHYPNEA |
| 78609 RESPIRATORY ABNORM NEC |
| 7862 COUGH |
| 78650 CHEST PAIN NOS |
| 78651 PRECORDIAL PAIN |
| 78652 PAINFUL RESPIRATION |
| 78659 CHEST PAIN NEC |
| 7931 ABN FINDINGS-LUNG FIELD |
