# Supplementary material for: Acute respiratory infection emergency access in a tertiary care children hospital in Italy, prior and after the SARS‐CoV‐2 emergence
Source: Influenza Other Respir Viruses. 2023 Mar 20;17(3):e13102. doi: 10.1111/irv.13102 (PMC10026100; doi:10.1111/irv.13102)
Supplement: Supplementary file 2 — Supplementary Material 2: Percentages of ARI ED virus attributed to different respiratory virus counts by age class [file IRV-17-e13102-s002.docx]

**Supplementary Material 2:** Percentages of ARI ED virus attributed to different respiratory virus counts by age class

| **Respiratory virus** | **Total population** | **<1 yr** | **1 - 4 yrs** | **5 - 9 yrs** | **>= 10 yrs** |
| --- | --- | --- | --- | --- | --- |
| **Adenovirus** | 8% | 10% | 8% | 11% |  |
| **RSV** | 6% | 20% | 6% |  |  |
| **Coronavirus** | 5% |  | 7% |  |  |
| **Metapneumovirus** | 5% | 7% | 4% | 7% |  |
| **Influenza** | 4% | 3% | 3% | 10% | 4% |
| **Parainfluenza** | 6% |  | 13% |  |  |
| **Rhinovirus** | 14% | 15% | 10% | 11% | 15% |
| **SARs-Cov2** | 3% |  | 4% |  | 7% |
| **Unattributed** | 49% | 44% | 46% | 61% | 74% |
